# B-Mode and Contrast-Enhanced Ultrasonography Aspects of Benign and Malignant Superficial Neoplasms in Dogs: A Preliminary Study

**DOI:** 10.3390/ani12202765

**Published:** 2022-10-14

**Authors:** Amber Hillaert, Emmelie Stock, Luc Duchateau, Hilde de Rooster, Nausikaa Devriendt, Katrien Vanderperren

**Affiliations:** 1 Department of Morphology, Imaging, Orthopedics, Rehabilitation and Nutrition, Faculty of Veterinary Medicine, Ghent University, 9820 Merelbeke, Belgium; 2 Department of Comparative Physiology and Biometry, Faculty of Veterinary Medicine, Ghent University, 9820 Merelbeke, Belgium; 3 Small Animal Department, Faculty of Veterinary Medicine, Ghent University, 9820 Merelbeke, Belgium; 4 Cancer Research Institute Ghent (CRIG), Medical Research Building, University Hospital Ghent, 9000 Ghent, Belgium

**Keywords:** contrast-enhanced ultrasound, B-mode ultrasound, perfusion, tumor, canine

## Abstract

**Simple Summary:**

In dogs, superficial neoplasms are common, and it is crucial to determine their malignancy, as this will have an impact on treatment and prognosis. So far, the diagnostic value of ultrasound modalities, such as B-mode and contrast enhanced ultrasound, for superficial neoplasms in dogs is still unclear, despite promising studies in humans. B-mode ultrasound enables assessment of the size, shape and arrangement of the neoplastic tissue, whereas contrast enhanced ultrasound enables the assessment of blood flow intensity and pattern. The aim of this study was to identify B-mode and contrast enhanced ultrasound characteristics that may be used to distinguish benign and malignant superficial neoplasms in dogs. Ultrasonographic characteristics, for which a significant difference was observed between benign and malignant neoplasms, were border definition, echogenicity, echotexture, blood flow pattern at wash-in and blood flow intensity during wash-out at the center of the neoplasm. Despite these significant differences, there was a considerable overlap in ultrasonographic characteristics between benign and malignant neoplasms. In conclusion, B-mode and contrast enhanced ultrasound might contribute to malignancy prediction; however, based on individual ultrasonographic characteristics, they seem unable to replace cytology or histopathology.

**Abstract:**

Contrast-enhanced ultrasonography (CEUS) is considered a promising technique for differentiation of benign and malignant tumors in humans. However, few studies have assessed superficial neoplasms in dogs by means of CEUS. The aim of this study was to identify ultrasonographic criteria evaluated by B-mode ultrasound (US) and CEUS that may be used to distinguish benign and malignant superficial neoplasms in dogs. A total of 63 superficial neoplasms from 59 dogs were evaluated using B-mode US and CEUS prior to histopathologic examination. Qualitative and quantitative parameters were compared between benign and malignant neoplasms by Fischer’s exact test or fixed effects model. With B-mode US, a significant difference was found for border definition, echogenicity and echotexture. With CEUS, a significant difference was found for the enhancement pattern at wash-in and the wash-out area under the curve at the center of the neoplasm. Malignant neoplasms had on average a lower regional blood volume during the wash-out phase compared to benign neoplasms. Despite these significant differences, there was a considerable overlap in B-mode and CEUS parameters between benign and malignant neoplasms. In conclusion, B-mode US and CEUS might contribute to malignancy prediction; however, based on individual ultrasonographic parameters, they seem unable to replace cytology or histopathology.

## 1. Introduction

In oncology, an accurate diagnosis by clinical assessment, followed by histological examination, is of great importance for the prognosis and selection of therapeutic approaches [1]. Staging is a way of describing the extent of a cancer in the body. It is based on neoplasm size, invasion into neighboring tissue, involvement of regional lymph nodes, and the presence of distant metastases [2]. Several imaging modalities may be used as non-invasive staging methods, as they may provide important information on neoplastic characteristics [3].

Skin, soft tissue and mammary glands are some of the most common locations for canine cancer development and are extremely suitable for ultrasonographic examination [4,5,6]. Ultrasonography (US) has advanced in recent years and its application as a complementary diagnostic technique in oncology is increasingly being investigated [7]. Two common techniques are B-mode and Doppler US, which allow non-invasive assessment of tissue morphology and vascularization, respectively [8]. Ultrasonographic characteristics seem to be related to histopathologic alterations [9] and are believed to potentially play a role in malignancy prediction [10]. Studies suggest that echotexture, border shape and vascular flow pattern, among others, either alone or combined, could aid in the assessment of malignancy and histological type (i.e., classification based on the tissue type from which the neoplasm originated) [10,11,12,13,14,15]. Ultrasonography would allow malignancy prediction with moderate accuracy [10]; about 75% of superficial tumors could be correctly classified by B-mode and Doppler US [10].

An emerging imaging modality for tumor assessment is contrast-enhanced US (CEUS). This is an ultrasound technique that enables real-time evaluation of blood flow and vasculature, including micro-vasculature, by intravenous administration of an ultrasound contrast agent [16,17]. Ultrasound contrast agents consist of tiny gas bubbles enclosed by an outer shell with an albumin, lipid or polymer composition [16,17]. Due to their size, ranging from 1–10 µm, the microbubbles are confined to the intravascular space [16,17]. CEUS has previously been used to monitor the effect of vascular disruptive therapy [18,19] and pharmacological agents [20] on tumor perfusion in dogs.

However, the diagnostic value of CEUS for superficial neoplasms in dogs is still unclear, as this technique has rarely been used to evaluate these. To our knowledge, only a preliminary study assessing the vascularity and perfusion of 34 superficially located tumors in dogs has been performed [21]. Other studies have focused specifically on the ability of CEUS to predict malignancy and histologic type of mammary tumors [14,22]. In the latter studies, CEUS, just like B-mode and Doppler US, shows an insufficient accuracy in discriminating between malignant and benign canine mammary tumors [14,22]. For a definitive diagnosis, invasive procedures, such as fine-needle aspirate cytology or biopsy, remain essential [1,13,23].

The aim of this study was to identify ultrasonographic criteria evaluated by B-mode US and CEUS that may be used to distinguish benign and malignant superficial neoplasms in dogs. B-mode US was used to visualize morphology, whereas CEUS was used to image vascularization, including perfusion type (centripetal, centrifugal, combined, chaotic) and perfusion pattern (heterogeneous, homogeneous, rim).

## 2. Materials and Methods

Data of solid neoplastic masses from client-owned dogs presented to the Small Animal Teaching Hospital of the Faculty of Veterinary Medicine of Ghent University (Merelbeke, Belgium) between 2013 and 2017 were included. Approval from the local research Ethical Committee (approval no. EC2013/32, EC2014/81, EC2015/143, EC2015/124, EC2016/66) of the Faculty of Veterinary Medicine of Ghent University, Belgium and from the Deontological Committee of the Federal Public Service of Health, Food Chain Safety and Environment, Belgium was obtained. Written informed consent was obtained from all patient owners before entry into the original study. The data have in part been used in previous publications (Cicchelero et al., 2017 [24], Cicchelero et al., 2017 [25], Abma et al., 2018 [18], Favril et al., 2020a [26] and Favril et al., 2020b [27]). Dogs were eligible for the original studies when the neoplasm was accessible with a linear ultrasound probe and the origin of the neoplasm was confirmed by histopathology. Dogs with abnormalities in cardiovascular parameters on physical examination (cardiac auscultation, blood pressure measurement) were excluded. At the time of presentation, the dogs were screened for the presence of metastases through thoracic radiographs and abdominal ultrasound.

The neoplastic masses were imaged using a Philips iU22 xMatrix ultrasound unit (Philips Medical systems, Bothell, Washington, DC, USA). Hair covering the mass was clipped and coupling gel (Aquasonic 100, Parker, Fairfield, NJ, USA) was applied. First, B-mode US was performed with a linear probe of 12–5 MHz or 17–5 MHz to define the neoplasm’s appearance. Next, the vascularity was assessed with CEUS using a linear 12–5 MHz probe. The mechanical index was set at 0.09; persistence was disabled; a single focus zone was placed under the lesion. If necessary, the dogs were sedated with butorphanol (Dolorex, 0.2 mg/kg), an opioid analgesic with no significant influence on renal CEUS parameters [28]. The contrast agent consisted of sulfur hexafluoride gas stabilized by a phospholipid membrane (Sonovue, Bracco, Milan, Italy) and was administered as a bolus (0.04 mL/kg) via a cephalic catheter (22 G). Subsequently, 1 mL of sterile physiological solution (Mini-Plasco NaCl 0.9%, Braun, Melsungen, Germany) was injected. Simultaneously with injection, a recording started and ran for 60 to 90 s. This procedure was repeated 2 to 3 times for each neoplasm. In between recordings, the remaining microbubbles in the neoplasm were removed by scanning the region at a high mechanical index. Clips were recorded and analyzed by an ECVDI Specialist with over 5 years of experience in CEUS and a PhD in the topic (E. S.).

The assessed B-mode ultrasonographic features included shape, delineation (well-defined, ill-defined), echogenicity in relation to the surrounding normal tissue (hyperechoic, hypoechoic, isoechoic, mixed), echotexture (homogeneous, heterogeneous), edge shadowing (yes, no) and the presence of cystic lesions and mineralization (yes, no).

Quantitative analysis of the perfusion was performed using a specialized software application (VueBox^®^ v6.2.0.55291, Bracco Suisse SA, Planles-Ouates, Switzerland). Three regions of interest (ROI) were manually drawn in each neoplasm, which comprised the entire neoplasm, the center, or the periphery. The program generated mean pixel intensities, which were plotted over time to create time-intensity curves (TIC) for every ROI. The following quantitative parameters were computed based on the time-intensity curves: intensity related parameters (peak enhancement (PE), wash-in area under the curve (WiAUC), wash-out area under the curve (WoAUC), wash-in wash-out area under the curve (WiWoAUC)), time-related parameters (time-to-peak (TTP), rise time (RT), fall time (FT), mean transit time (mTTI)) and slope related parameters (wash-in perfusion index (WiPI), wash-in rate (WiR), and wash-out rate (WoR)). Wash-in and wash-out refer to the portions of the TIC before and after the enhancement peak, respectively.

The qualitative parameters used to subjectively assess the vascularity and blood flow by CEUS were wash-in (centripetal, centrifugal, chaotic, combined), enhancement pattern at wash-in, peak and wash-out (homogeneous, heterogeneous, rim), enhancement degree in relation to adjacent healthy tissue at wash-in, peak and wash-out (hyperechoic, hypoechoic, isoechoic) and the presence of non-perfused areas and large vessels (yes, no).

Histopathological evaluation of the resected neoplasms was performed as described by Cicchelero et al., 2017 [24], Cicchelero et al., 2017 [25], Abma et al., 2018 [18], Favril et al., 2020a [26] and Favril et al., 2020b [27]. The histological assessment was conducted by a board-certified veterinary pathologist. Based on these findings, the masses were classified into benign neoplasms or malignant neoplasms. As grade I mastocytomas, labelled according to the Patnaik system, have been associated with a low metastatic risk and good-to-excellent prognosis [29,30,31,32], they were included in the group of benign neoplasms.

Analysis was conducted in R version 4.1.1. A fixed effects model was fitted to assess the effect of benign classification on quantitative CEUS parameters for all superficial neoplasms using F-tests at the 5% significance level (R lm function). The effect of benign classification on B-mode and qualitative CEUS parameters (binary and categorical) was analysed using Fischer’s exact tests (R fisher.test function). In addition, the same analyses were performed exclusively on mammary gland neoplasms.

Extra analyses were performed in which grade I carcinomas were not included. This is because the metastatic potential, which determines malignancy, associated with grade I is limited.

## 3. Results

### 3.1. Study Population

Data from 63 neoplasms were included in the study. Thirty-six neoplasms were located at the level of the mammary gland and 27 were located at various superficial locations: head (*n* = 1), nose (*n* = 1), maxilla (*n* = 1), mandible (*n* = 1), intra-orally (*n* = 3), front limb (*n* = 8), hind limb (*n* = 5), rib (*n* = 2), abdomen (*n* = 2), anal gland (*n* = 1), anal sac (*n* = 1), and perianal (*n* = 1). Based on histopathological examination, the neoplasms were divided into neoplastic benign (*n* = 16) and neoplastic malign (*n* = 47).

The neoplasms belonged to 59 dogs of 27 different breeds, 42 were female (25 intact, 17 spayed) and 17 male (4 intact and 13 neutered). The mean ± standard deviation age of the dogs included in the study was 9.5 ± 3.17 years and ranged from 2 to 17 years. A summary of the histologic types included in the benign and malignant neoplastic group is presented in Table 1.

### 3.2. Imaging Results

#### 3.2.1. B-Mode Ultrasound

A significant effect of classification was observed on border definition (*p* = 0.04), echogenicity (*p* = 0.001) and echotexture (*p* = 0.04). When grade one carcinomas were not included in the analysis, a significant effect was also observed on shape (*p* = 0.02). The ultrasonographic results obtained by B-mode US are summarized in Table 2.

#### 3.2.2. Qualitative CEUS

A significant effect of classification was only observed on the enhancement pattern at wash-in (*p* = 0.047). The results of qualitative CEUS characteristics are summarized in Table 3.

**Figure 1:**
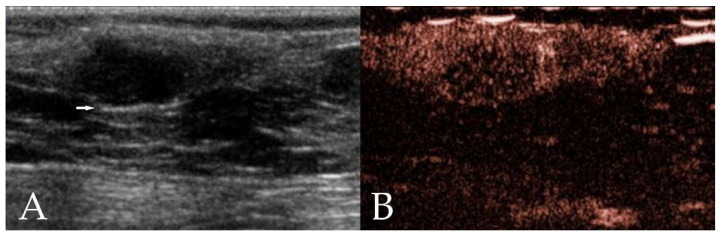
Ultrasonographic images of a mammary adenoma in a 12 year old intact Border Collie. (**A**) On B-mode ultrasonography, the neoplasm shows an ovoid shape, well-defined border, hypoechoic homogeneous echotexture, and the presence of edge enhancement (arrow). (**B**) Contrast-enhanced ultrasonography (CEUS) shows heterogeneous enhancement.

**Figure 2:**
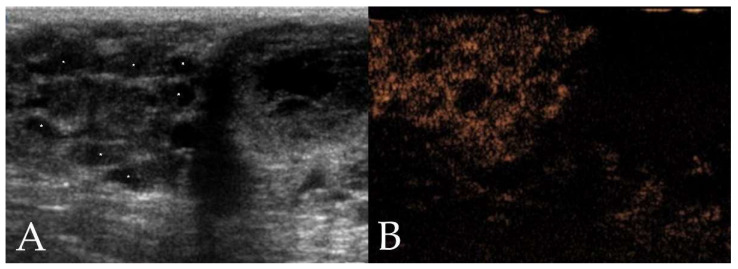
Ultrasonographic images of a mammary adenocarcinoma in a 10 year old intact Labrador Retriever. (**A**) On B-mode ultrasonography, the neoplasm shows a multilobulated shape, ill-defined border, mixed echogenicity, and heterogeneous echotexture with presence of cystic areas (stars). (**B**) Contrast-enhanced ultrasonography (CEUS) shows heterogeneous enhancement.

Benign neoplasms mainly showed an ovoid shape, well-defined border, hypoechoic echogenicity, homogeneous echotexture and a centripetal pattern at wash-in (Figure 1). Malignant neoplasms mainly had an multilobulated or ovoid shape, well- or ill-defined border, mixed echogenicity, heterogeneous echotexture and a chaotic pattern at wash-in (Figure 2).

#### 3.2.3. Quantitative CEUS

A significant effect of classification was observed on WoAUC (*p* = 0.046) at the center of the neoplasm. Compared with benign neoplasms, malignant neoplasms had a significantly smaller AUC during the wash-out phase, which indicates there is less regional blood volume. The mean WoAUC in benign neoplasms was approximately double compared to malignant neoplasms. Table A1 in Appendix A shows an overview of the mean WoAUC at the center of the neoplasm for each histological type of neoplasm.

## 4. Discussion

The current study found that benign and malignant neoplasms differed significantly in certain ultrasonographic characteristics determined by B-mode US and CEUS, suggesting these could contribute to malignancy prediction. For B-mode US, this involved border definition, echogenicity and echotexture. For CEUS, this concerned the qualitative parameter enhancement pattern at wash-in and quantitative parameter WoAUC. Benign neoplasms mainly showed an ovoid shape, well-defined border, hypoechoic echogenicity, homogeneous echotexture and a centripetal pattern at wash-in. Malignant neoplasms mainly had a multilobulated or ovoid shape, well- or ill-defined border, mixed echogenicity, heterogeneous echotexture and a chaotic pattern at wash-in.

Tissue morphology is essential in the differentiation of malignant and benign neoplasms. Non-invasive assessment of tissue morphology is, to some extent, feasible with B-mode-US, as ultrasonographic observations have been correlated with histopathological findings [9,33]. The potential of US in malignancy prediction of superficial tumors has been reported by several authors [10,13,22]. At present, ultrasound examination might assist in a first evaluation; however, it is not able to replace cytology or histopathology [10,13,22]. Moreover, malignancy prediction is based on vascularity [34,35,36,37]. Neo-angiogenesis, i.e., new blood vessel formation, in neoplasms is induced by metabolic insufficiency and mechanical stress [38,39]. In contrast to normal vascularisation, neoplastic blood supply shows an abnormal function, structure and organization [38]. Neoplastic blood vessels have a tortuous course, an enlarged, irregular diameter and are unequally and excessively branched [38,39]. Non-invasive vascular assessment is possible with Doppler US and CEUS [22,40]. Similar to B-mode US, Doppler US might assist in the prediction of malignancy, but it is unable to replace histopathological examination [22,41]. This may be due to the fact that the ability of Doppler US to detect tissue perfusion is limited and depends on blood vessel size, among others [42]. Vessels have to be at least 100 µm in diameter to be detected by Doppler US [40,43]. In contrast, CEUS enables the visualization of vessels with a diameter of 40 µm [44] and approximates histological analysis, by which blood vessels of 15 µm can be detected [40,43]. In humans, CEUS is considered a promising technique for the differentiation of benign and malignant superficial lesions [45]. However, few studies have assessed superficial neoplasms in dogs by means of CEUS [22,41].

Prior studies have reported that ill-defined margins seen with B-mode US are suggestive of invasive growth and are an important indicator of malignancy [46,47]. Well-defined margins, on the other hand, have previously been associated with benign tumors [46,48]. This is in line with the current study which found that border definition was significantly different between benign and malignant neoplasms. Benign neoplasms generally showed a well-defined border in a greater percentage of cases than malignant neoplasms. Contrarily, according to a number of studies on superficial tumors [10] and mammary tumors [9,11,13,22,49] in dogs, border definition was not statistically different. However, a trend could sometimes be observed. In two studies, for instance, malignant tumors more frequently showed ill-defined margins in comparison to benign tumors [9,13].

With regard to the use of echogenicity as prognostic parameter, previous studies reported inconsistent results. While some authors found no correlation with malignancy of canine mammary tumors [11,22,49,50], other authors observed a significant difference in echogenicity between benign and malignant tumors [9,10,48]. According to these authors, benign tumors were more likely being isoechoic or hypoechoic, whereas malignant tumors were more likely to have a mixed echogenicity. Similar results were observed in the current study, with hypoechogenic echogenicity being the most frequently observed in benign neoplasms and mixed echogenicity being the most frequently observed in the malignant neoplasms.

Echotexture has been identified as a helpful ultrasonographic criteria in the differentiation of benign and malignant mammary masses in both dogs [9,11,13] and humans [51,52]. A homogeneous echotexture has been related to benignancy [13,52], while a heterogeneous echotexture has been related to malignancy [9,11,13,51]. Histologically, a heterogeneous echotexture is correlated with the presence of diverse tissue components, such as necrosis, hemorrhage and cysts [9,41]. In malignant neoplasms, central necrosis regularly arises due to hypoxia and insufficient nutrient supply associated with rapid growth [53,54]. In addition, in the current study, the majority of the malignant neoplasms had a heterogeneous appearance, while a homogeneous echotexture was mainly observed in benign neoplasms. A few studies of canine mammary masses, however, reported echotexture to be an ineffective indicator of malignancy [14,41].

Perfusion pattern at wash-in is another parameter, which was significantly different between benign and malignant neoplasms in this study. Benign neoplasms mainly presented a centripetal pattern at wash-in, while a chaotic vascular pattern was mostly observed in malignant neoplasms. In canine mammary tumors, no significant difference was found in the perfusion pattern, but the majority of malignancies showed a centrifugal pattern, whereas the majority of benign tumors had a diffuse pattern [22]. In humans, malignant breast lesions were mainly characterized by a centripetal pattern, whereas benign breast lesions were mainly characterized by a centrifugal pattern [45,55,56].

With CEUS, non-perfused areas correspond to hypoechoic regions and are presumed to be hypoxia-induced necrosis [57,58]. Perfusion defects have been reported as a promising prognostic criterion in studies of human breast cancer [55,59]. Moreover, perfusion patterns characterized by non-enhancing areas have been associated with malignancy [59,60], while homogeneous or complete non-enhancing perfusion patterns have been related with benignancy [60,61]. In superficial canine tumors and mammary canine tumors, however, no significant difference in vascular pattern has been observed between groups [10,22]. Also in this study, no significant differences were found between benign and malignant neoplasms for the presence of non-perfused areas.

Edge shadowing is an artifact that typically occurs when circular structures are imaged on ultrasound [62,63]. It is characterized by hypoechoic to anechoic regions posterior to the curved edges [62,63]. As this artifact is correlated with a morphologic characteristic of the lesion, it may be helpful in the evaluation of suspicious masses [64]. In the present study, no significant difference between benign and malignant neoplasms was observed for edge shadowing. In a previous study on canine mammary tumors, nearly 90% of benign masses showed edge shadowing, while this was absent in all malignant masses [64].

Enhancement degree compared to surrounding tissue represents the relative blood supply of a lesion. At various vascular phases, it has been shown to possibly improve differentiation of benign and malignant breast tumors [45,55]. In humans, hyperechogenicity at the peak time was suggestive of breast cancer malignancy [45,55]. In canine mammary tumors, no significant difference was found, but malignant mammary gland tumors frequently showed a lower level of enhancement than benign lesions [22]. In the present study, no significant difference was found between benign and malignant neoplasms for enhancement degree, despite more than half of the samples being mammary gland neoplasms. Even when the enhancement degree of the mammary gland neoplasms was analyzed separately, no significant differences were observed. Furthermore, the percentage of neoplasms with high enhancement levels was greater in malignant than benign mammary neoplasms.

Regarding quantitative parameters determined by CEUS, WoAUC in the center of the tumor was identified as a potential valuable ultrasonographic characteristic for malignancy prediction in this study. Benign neoplasms had a higher WoAUC than malignant neoplasms. In addition, benign neoplasms had a tendency to have higher values compared to malignant neoplasms for FT (*p* = 0.09), WiAUC (*p* = 0.09), WiWoAUC (*p* = 0.059) at the center of the neoplasm and for WoAUC (*p* = 0.08) in the region of interest compromising the entireneoplasm. However, statistically, these differences could not be demonstrated to be significant. In dogs, only a limited number of studies have performed CEUS quantitative analysis. One study reported that no quantitative parameter significantly correlated with malignancy in canine mammary tumors [22]. Another study found that longer RT, TTP and FT were suggestive of high-grade mammary carcinomas [14]. In breast lesions of humans, multiple quantitative parameters have been shown to significantly differ between malignant and benign masses [55,65]. For example, significant differences were observed for TTP, PE and AUC [55,65]. These findings mainly indicated a higher blood velocity and greater blood flow in malignant masses, which was attributed to their abnormal vascular architecture and increased vessel density due to angiogenesis [55,65].

The main limitation of this study is the low number of benign neoplasms. A larger sample size may have yielded more significant results. A second limitation is that the majority of the superficial neoplasms were mammary gland neoplasms resulting in a less heterogenous population of superficial neoplasms. Additionally, the combined use of ultrasonographic characteristics in malignancy prediction was not explored. Considering the lymphatic system’s importance for metastasis, future research should also include the draining lymph nodes and investigate the potential role of CEUS in the detection of metastases [66].

This study identified B-mode US and CEUS parameters that might play a role in malignancy prediction of neoplasms. Nonetheless, given the overlap in these ultrasonographic parameters between benign and malignant neoplasms, differentiation based on these individual ultrasonographic parameters seems unreliable. Previous studies have shown, however, that the combination of several ultrasound criteria, which in themselves have little diagnostic value, allows for malignancy prediction and tumor type identification with moderate accuracy [10,12,14,45,61]. Therefore, combined with known parameters of interest, newly identified parameters from this study might result in improved diagnosis of neoplasms. 

## 5. Conclusions

In conclusion, B-mode US and qualitative CEUS might contribute to malignancy prediction. Potentially valuable B-mode US and qualitative CEUS parameters that have been identified are border definition, echogenicity, echotexture and enhancement pattern at wash-in. A quantitative CEUS parameter which has been identified with potential value in malignancy prediction is WoAUC at the center of the tumor. Notwithstanding the relatively limited sample, these findings suggest that US may have an important role in the evaluation of superficial neoplasms in dogs. Further studies using a larger sample size are needed to specify the possibilities of US in cancer evaluation. More specifically, the combined use of ultrasonographic characteristics in malignancy prediction should be explored.

## Figures and Tables

**Table 1:** Histologic types of the benign and malignant superficial neoplasms evaluated in dogs.

	Histological Type	Origin	Number
Benign	Adenoma	Mammary gland	11
	Complex adenoma	Mammary gland	4
	Mastocytoma Grade I	Hind leg	1
Total			16
Malignant	Complex adenocarcinoma Grade I	Mammary gland	2
	Adenocarcinoma Grade I	Mammary gland	7
	Adenocarcinoma Grade II	Mammary gland	2
	Adenocarcinoma Grade III	Mammary gland	3
	Adenocarcinoma NG	Anal gland, anal sac	2
	Mastocytoma Grade II	Abdomen (2), hind leg (3)	5
	Mastocytoma Grade III	Front limb, hind limb	2
	Mastocytoma NG	Head	1
	Fibrosarcoma	Intra-oral (1), maxilla (1), front limb (2)	4
	Neurofibrosarcoma	Peri-anal	1
	Schwannoma	Front leg	4
	Osteosarcoma	Rib (2), intra-oral (1)	3
	Extra skeletal osteosarcoma	Mammary gland	2
	Inflammatory carcinoma	Mammary gland	2
	Squamous cell carcinoma	Mammary gland, mandible	2
	Cystadenocarcinoma	Mammary gland	1
	Complex carcinoma	Mammary gland	1
	Melanoma	Intra-oral	1
	Chondrosarcoma	Front limb	1
	Histiocytic sarcoma	Nose	1
Total			47

NG: not graded.

**Table 2:** An overview of ultrasonographic characteristics obtained by B-mode ultrasonography for benign and malignant neoplasms.

	Benign Neoplastic	Malignant Neoplastic	*p*-Value
**Shape**			0.11
Ovoid	75.0%	38.3%	
Round	6.3%	10.6%	
Multilobulated	12.5%	40.4%	
Irregular	6.3%	8.5%	
Other	0.0%	2.1%	
**Border definition**			**0.04**
Well-defined	81.0%	48.9%	
Ill-defined	19.0%	51.1%	
**Echogenicity**			**0.01**
Hyperechoic	12.5%	2.1%	
Hypoechoic	56.3%	27.7%	
Isoechoic	18.7%	19.1%	
Mixed	12.5%	51.1%	
**Echotexture**			**0.04**
Heterogeneous	37.5%	68.1%	
Homogeneous	62.5%	31.9%	
**Edge shadowing**			0.06
Present	37.5%	12.8%	
Absent	62.5%	87.2%	
**Cystic areas**			0.74
Present	25.0%	21.3%	
Absent	75.0%	78.7%	
**Mineralization**			0.35
Present	19.0%	34.0%	
Absent	81.0%	66.0%	

Characteristics for which a significant effect (*p* < 0.05) was observed are in bold.

**Table 3:** An overview of vascular ultrasonographic characteristics obtained by contrast-enhanced ultrasonography for benign and malignant neoplasms.

	Benign Neoplastic	Malignant Neoplastic	*p*-Value
**Wash-in**			**0.05**
Centripetal	68.7%	31.9%	
Centrifugal			
Combined		2.1%	
Chaotic	31.3%	57.4%	
NE		8.5%	
**Large vessels**			0.32
Present	12.5%	25.5%	
Absent	87.5%	70.2%	
NE		4.3%	
**Non-perfused areas**			0.25
Present	37.5%	53.2%	
Absent	62.5%	42.6%	
**Enhancement at wash-in**			1.00
Heterogeneous	93.7%	83%	
Rim	6.3%	8.5%	
Homogeneous			
NE		8.5%	
**Enhancement at peak**			0.26
Heterogeneous	75%	78.7%	
Rim	6.3%	8.5%	
Homogeneous	18.7%	4.3%	
NE		8.5%	
**Enhancement at wash-out**			1.00
Heterogeneous	93.7%	83%	
Rim	6.3%	8.5%	
Homogeneous			
NE		8.5%	
**Enhancement degree at wash-in**			0.12 *
Hyperechoic	43.8%	36.2%	
Hypoechoic	18.8%	23.4%	
Isoechoic	19%	2%	
NE	18.8%	38.3%	
**Enhancement degree at peak**			0.31 *
Hyperechoic	38%	36%	
Hypoechoic	18.8%	19%	
Isoechoic	25%	6.4%	
NE	18.8%	38.3%	
**Enhancement degree at wash-out**			0.22 *
Hyperechoic		8.5%	
Hypoechoic	43.8%	17%	
Isoechoic	37.5%	36.2%	
NE	18.8%	38.3%	

Characteristics for which a significant effect (*p* < 0.05) was observed are in bold. * 33% of the data was missing. NE: perfusion was not evaluable.

## Data Availability

Upon request, the data presented in this study can be provided by the corresponding author.

## Appendix A

**Table A1:** A summary of the mean wash-out area under the curve (WoAUC) at the center of the neoplasm according to histological type.

Histological Type	Number	Mean WoAUC (a.u.)
**Benign neoplasms**		
Adenoma	11	15,442
Complex adenoma	4	10,443
Mastocytoma Grade I	1	1167
**Malignant neoplasms**		
Complex adenocarcinoma Grade I	2	9677
Adenocarcinoma Grade I	7	5016
Adenocarcinoma Grade II	2	7488
Adenocarcinoma Grade III	3	13,688
Adenocarcinoma NG	2	6931
Mastocytoma Grade II	5	2247
Mastocytoma Grade III	2	7539
Mastocytoma NG	1	8314
Fibrosarcoma	4	4156
Neurofibrosarcoma	1	807
Schwannoma	4	3207
Osteosarcoma	3	4778
Extra skeletal osteosarcoma	2	533
Inflammatory carcinoma	2	13,541
Squamous cell carcinoma	2	2307
Cystadenocarcinoma	1	14,820
Complex carcinoma	1	1491
Melanoma	1	3290
Chondrosarcoma	1	96
Histiocytic sarcoma	1	1675

a.u.: arbitrary units.

## References

[1] Dobson J.M., Lascelles B.D. (2011). BSAVA Manual of Canine and Feline Oncology.

[2] Kamstock D.A., Russell D.S., Powers B.E., Vail D.M., Thamm D.H., Liptak J.M. (2019). The pathology of neoplasia. Withrow and MacEwen’s Small Animal Clinical Oncology-E-Book.

[3] Nykamp S., Randall E., Vail D.M., Thamm D.H., Liptak J.M. (2019). Diagnostic imaging in oncology. Withrow and MacEwen’s Small Animal Clinical Oncology-E-Book.

[4] Dobson J.M., Samuel S., Milstein H., Rogers K., Wood J.L.N. (2002). Canine neoplasia in the UK: Estimates of incidence rates from a population of insured dogs. J. Small Anim. Pract..

[5] Merlo D.F., Rossi L., Pellegrino C., Ceppi M., Cardellino U., Capurro C., Ratto A., Sambucco P.L., Sestito V., Tanara G. (2008). Cancer Incidence in Pet Dogs: Findings of the Animal Tumor Registry of Genoa, Italy. J. Vet. Intern. Med..

[6] Brønden L.B., Nielsen S.S., Toft N., Kristensen A.T. (2010). Data from the Danish veterinary cancer registry on the occurrence and distribution of neoplasms in dogs in Denmark. Vet. Rec..

[7] Wisner E.R., Pollard R.E. (2004). Trends in veterinary cancer imaging. Vet. Comp. Oncol..

[8] Mattoon J.S., Nyland T.G., Mattoon J.S., Nyland T.G. (2015). Fundamentals of diagnostic ultrasound. Small Anim Diagnostic Ultrasound Mattoon.

[9] Nyman H.T., Nielsen O.L., McEvoy F.J., Lee M.H., Martinussen T., Hellmén E., Kristensen A.T. (2006). Comparison of B-mode and Doppler ultrasonographic findings with histologic features of benign and malignant mammary tumors in dogs. Am. Coll. Vet. Radiol..

[10] Nyman H.T., Kristensen A., Lee M.H., Martinussen T., McEvoy F.J. (2006). Characterization of canine superficial tumors using gray-scale B mode, color flow mapping, and spectral doppler ultrasonography-A multivariate study. Vet. Radiol. Ultrasound.

[11] Brandão Guedes P.E., Bandeira Thame Daniel H., Oliveira Rosa Sampaio K.M., Barboza da Silva E., Ferreira M.L., Lima de Lavor M.S., De Oliveira Clark R.M., Wenceslau A.A., Abou Said R., Lessa Silva F. (2020). Clinical and Ultrasonographic Aspects of Benign and Malignant Mammary Tumors in Female Dogs. Acta Sci. Vet..

[12] Loh Z.H.K., Allan G.S., Nicoll R.G., Hunt G.B. (2009). Ultrasonographic characteristics of soft tissue tumours in dogs. Aust. Vet. J..

[13] Soler M., Dominguez E., Lucas X., Novellas R., Gomes-coelho K.V., Espada Y., Agut A. (2016). Comparison between ultrasonographic findings of benign and malignant canine mammary gland tumours using B-mode, colour Doppler, power Doppler and spectral Doppler. Res. Vet. Sci..

[14] Feliciano M.A.R., Ramirez A.R.U., Maronezi M.C., Maciel R.A., Avante M.L., Senhorello I.L.S., Mucédola T., Gasser B., Carvalho C.F., Vicente W.R.R. (2018). Accuracy of four ultrasonography techniques in predicting histopathological classification of canine mammary carcinomas. Am. Coll. Vet. Radiol..

[15] Balaci I.M., Ciupe S., Pop A.R., Parlapan L., Arion A., Vidrighinescu R., Groza I.S. (2014). Ultrasonographig Aspects of Mammary Tumours in Bitches. Bull. UASVM Vet. Med..

[16] Pietsch H., Kiessling F., Pichler B.J., Hauf P. (2011). Ultrasound contrast agents. Small Animal Imaging.

[17] Correas J.-M., Bridal L., Lesavre A., Méjean A., Claudon M., Hélénon O. (2001). Ultrasound contrast agents: Properties, principles of action, tolerance, and artifacts. Eur. Radiol..

[18] Abma E., De Spiegelaere W., Vanderperren K., Stock E., Van Brantegem L., Cornelis I., Daminet S., Ni Y., Vynck M., Verstraete G. (2018). A single dose of intravenous combretastatin A4-phosphate is reasonably well tolerated and significantly reduces tumour vascularization in canine spontaneous cancers. Vet. Comp. Oncol..

[19] Abma E., Stock E., De Spiegelaere W., Brantegem L.V., Vanderperren K., Ni Y., Vynck M., Daminet S., Clercq K.D., de Rooster H. (2019). Power Doppler ultrasound and contrast-enhanced ultrasound demonstrate non-invasive tumour vascular response to anti-vascular therapy in canine cancer patients. Sci. Rep..

[20] Quartuccio M., Mangano C., Macri F., Rizzo M., Di Pietro S., Pugliese M., Mazzullo G., Cristarella S., De Majo M. (2018). Contrast-enhanced ultrasound evaluation of testicular interstitial cell tumours in conscious non-sedated dogs. Vet. Med. (Praha).

[21] Schärz M., Ohlerth S., Achermann R., Gardelle O., Roos M., Saunders H.M., Wergin M., Kaser-Hotz B. (2005). Evaluation of quantified contrast-enhanced color and power Doppler ultrasonography for the assessment of vascularity and perfusion of naturally occuring tumors in dogs. Am. J. Vet. Res..

[22] Feliciano M.A.R., Uscategui R.A.R., Maronezi M.C., Simões A.P.R., Silva P., Gasser B., Pavan L., Carvalho C.F., Canola J.C., Vicente W.R.R. (2017). Ultrasonography methods for predicting malignancy in canine mammary tumors. PLoS ONE.

[23] Ramos-Vara J.A., Borst L.B., Meuten D.J. (2017). Immunohistochemistry: Fundamentals and applications in oncology. Tumors in Domestic Animals.

[24] Cicchelero L., Denies S., Vanderperren K., Stock E., Van Brantegem L., de Rooster H., Sanders N.N. (2017). Immunological, anti-angiogenic and clinical effects of intratumoral interleukin 12 electrogene therapy combined with metronomic cyclophosphamide in dogs with spontaneous cancer: A pilot study. Cancer Lett..

[25] Cicchelero L., Denies S., Haers H., Vanderperren K., Stock E., Van Brantegem L., de Rooster H., Sanders N.N. (2017). Intratumoural interleukin 12 gene therapy stimulates the immune system and decreases angiogenesis in dogs with spontaneous cancer. Vet. Comp. Oncol..

[26] Favril S., Abma E., Stock E., Devriendt N., Van Goethem B., Blasi F., Brioschi C., Polis I., De Cock H., Miragoli L. (2020). Fluorescence-guided surgery using indocyanine green in dogs with superficial solid tumours. Vet. Rec..

[27] Favril S., Brioschi C., Vanderperren K., Abma E., Devriendt N., Polis I., De Cock H., Cordaro A., Miragoli L., Olivia P. (2020). Preliminary safety and imaging efficacy of the near-infrared fluorescent contrast agent DA364 during fluorescence-guided surgery in dogs with spontaneous superficial tumors. Oncotarget.

[28] Stock E., Vanderperren K., Van Der Vekens E., Haers H., Duchateau L., Polis I., Hesta M., Saunders J.H. (2014). The effect of anesthesia with propofol and sedation with butorphanol on quantitative contrast-enhanced ultrasonography of the healthy feline kidney. Vet. J..

[29] Sledge D.G., Webster J., Kiupel M. (2016). Canine cutaneous mast cell tumors: A combined clinical and pathologic approach to diagnosis, prognosis, and treatment selection. Vet. J..

[30] Misdorp W. (2004). Mast cells and canine mast cell tumours. A review. Vet. Q..

[31] London C.A., Seguin B. (2003). Mast cell tumors in the dog. Vet. Clin. North Am. Small Anim. Pract..

[32] Patnaik A.K., Ehler W.J., MacEwen E.G. (1984). Canine Cutaneous Mast Cell Tumor: Morphologic Grading and Survival Time in 83 Dogs. Vet. Pathol..

[33] Ohlerth S., Wergin M., Bley C.R., Del Chicca F., Laluhová D., Hauser B., Roos M., Kaser-Hotz B. (2010). Correlation of quantified contrast-enhanced power Doppler ultrasonography with immunofluorescent analysis of microvessel density in spontaneous canine tumours. Vet. J..

[34] Vermeulen P.E., Gasparini G., Fox S.B., Toi M., Martin L., McCulloch P., Pezzella F., Viale G., Weidner N., Harris A.L. (1996). Quantification of angiogenesis in solid human tumours: An international consensus on the methodology and criteria of evaluation. Eur. J. Cancer Part A.

[35] Ranieri G., Passantino L., Patruno R., Passantino G., Jirillo F., Catino A., Mattioli V., Gadaleta C., Ribatti D. (2003). [Abstract] The dog mast cell tumour as a model to study the relationship between angiogenesis, mast cell density and tumour malignancy. Oncol. Rep..

[36] Preziosi R., Sarli G., Paltrinieri M. (2004). Prognostic Value of Intratumoral Vessel Density in Cutaneous Mast Cell Tumours of the Dog. J. Comp. Pathol..

[37] Diessler M.E., Castellano M.C., Portiansky E.L., Burns S., Idiart J.R. (2017). Canine mammary carcinomas: Influence of histological grade, vascular invasion, proliferation, microvessel density and VEGFR2 expression on lymph node status and survival time. Vet. Comp. Oncol..

[38] Carmeliet P., Jain R.K. (2000). Angiogenesis in cancer and other diseases. Nature.

[39] Nagy J.A., Chang S.-H., Shih S.-C., Dvorak A.M., Dvorak H.F. (2010). Heterogeneity of the Tumor Vasculature. Semin. Thromb. Hemost..

[40] Ohlerth S., Kaser-Hotz B. (2003). A review of Doppler sonography for the assessment of tumour vascularity. Vet. Comp. Oncol..

[41] Feliciano M.A.R., Vicente W.R.R., Silva M.A.M. (2012). Conventional and Doppler ultrasound for the differentiation of benign and malignant canine mammary tumours. J. Small Anim. Pract..

[42] Wilson S., Greenbaum L., Goldberg B. (2009). Contrast-enhanced ultrasound: What Is the Evidence and What Are the Obstacles?. Am. J. Roentgenol..

[43] Fleischer A.C., Wojcicki W.E., Donnelly E.F., Pickens D.R., Thirsk G., Thurman G.B., Hellerqvist C.G. (1999). Quantified color Doppler sonography of tumor vascularity in an animal model. J. Ultrasound Med..

[44] Tranquart F., Correas J.M., Ladam Marcus V., Manzoni P., Vilgrain V., Aube C., Elmaleh A., Chami L., Claudon M., Cuilleron M. (2009). Real-time contrast-enhanced ultrasound in the evaluation of focal liver lesions: Diagnostic efficacy and economical issues from a French multicentric study. J. Radiol..

[45] Wang Y., Fan W., Zhao S., Zhang K., Zhang L., Zhang P., Ma R. (2016). Qualitative, quantitative and combination score systems in differential diagnosis of breast lesions by contrast-enhanced ultrasound. Eur. J. Radiol..

[46] Mohammed S.I., Meloni G.B., Pinna Parpaglia M.L., Marras V., Burrai G.P., Meloni F., Pirino S., Antuofermo E. (2011). Mammography and ultrasound imaging of preinvasive and invasive canine spontaneous mammary cancer and their similarities to human breast cancer. Cancer Prev. Res..

[47] Zonderland H.M. (2000). The role of ultrasound in the diagnosis of breast cancer. Semin. Ultrasound CT MRI.

[48] Baştan A., Özenç E., Yaǧci I.P., Baki Acar D. (2009). Ultrasonographic evaluation of mammary tumors in bitches. Kafkas Univ. Vet. Fak. Derg..

[49] Vannozzi I., Tesi M., Zangheri M., Innocenti M.V., Rota A., Citi S., Poli A. (2018). B-mode ultrasound examination of canine mammary gland neoplastic lesions of small size (diameter <2 cm). Vet. Res. Commun..

[50] Tagawa M., Kanai E., Shimbo G., Kano M., Kayanuma H. (2016). Ultrasonographic evaluation of depth–width ratio (D/W) of benign and malignant mammary tumors in dogs. J. Vet. Med. Sci..

[51] Paulinelli R.R., Freitas-Júnior R., Moreira M.A.R., Alves de Moraes V., Bernardes-Júnior J.R.M., da Silva Rocha C., Naldi Ruiz A., Tostes Lucato M. (2005). Risk of malignancy in solid breast nodules according to their sonographic features. J. Ultrasound Med..

[52] Calas M.J.G., Koch H.A., Dutra M.V.P. (2007). Breast ultrasound: Evaluation of echographic criteria for differentiation of breast lesions. Radiol. Bras..

[53] Metz S., Daldrup-Link H.E., Richter T., Räth C., Ebert W., Settles M., Rummeny E.J., Link T.M., Piert M. (2003). Detection and Quantification of Breast Tumor Necrosis with MR Imaging: Value of the Necrosis-avid Contrast Agent Gadophrin-3. Acad. Radiol..

[54] Forster J.C., Harriss-phillips W.M., Douglass M.J.J., Bezak E. (2017). A review of the development of tumor vasculature and its effects on the tumor microenvironment. Hypoxia.

[55] Wan C., Du J., Fang H., Li F., Wang L. (2012). Evaluation of breast lesions by contrast enhanced ultrasound: Qualitative and quantitative analysis. Eur. J. Radiol..

[56] Wan C.F., Du J., Fang H., Li F.H., Zhu J.S., Liu Q. (2012). Enhancement Patterns and Parameters of Breast Cancers at Contrast-enhanced US: Correlation with Prognostic Factors. Radiology.

[57] Dunst J., Ahrens S., Paulussen M., Burdach S., Jürgens H. (2001). Prognostic impact of tumor perfusion in MR-imaging studies in Ewing tumors. Strahlentherapie Onkol..

[58] Nakamura K., Sasaki N., Murakami M., Kumara W.R.B., Ohta H., Yamasaki M., Takagi S., Osaki T., Takiguchi M. (2010). Contrast-Enhanced Ultrasonography for Characterization of Focal Splenic Lesions in Dogs. J. Vet. Intern. Med..

[59] Cao X.L., Bao W., Zhu S.G., Wang L.H., Sun M.H., Wang L., Men Y.M., Xue J. (2014). Contrast-enhanced ultrasound characteristics of breast cancer: Correlation with prognostic factors. Ultrasound Med. Biol..

[60] Loizides A., Peer S., Plaikner M., Djurdjevic T., Gruber H. (2012). Perfusion pattern of musculoskeletal masses using contrastenhanced ultrasound: A helpful tool for characterisation?. Eur. Radiol..

[61] Gruber L., Loizides A., Luger A.K., Glodny B., Moser P., Henninger B., Gruber H. (2017). Soft-tissue tumor contrast enhancement patterns: Diagnostic value and comparison between ultrasound and MRI. Am. J. Roentgenol..

[62] Hindi A., Peterson C., Barr R.G. (2013). Artifacts in diagnostic ultrasound. Reports Med. Imaging.

[63] Steel R., Poepping T.L., Thompson R.S., MacAskill C. (2004). Origins of the edge shadowing artefact in medical ultrasound imaging. Ultrasound Med. Biol..

[64] Gonzalez De Bulnes A., Garcia Fernandez P., Mayenco Aguirre A.M., Sanchez De la Muela M. (1998). Ultrasonographic imaging of canine mammary tumours. Vet. Rec..

[65] Caproni N., Marchisio F., Pecchi A., Canossi B., Battista R., D’Alimonte P., Torricelli P. (2010). Contrast-enhanced ultrasound in the characterisation of breast masses: Utility of quantitative analysis in comparison with MRI. Eur. Radiol..

[66] Collivignarelli F., Tamburro R., Aste G., Falerno I., Del Signore F., Simeoni F., Patsikas M., Gianfelici J., Terragni R., Attorri V. (2021). Lymphatic drainage mapping with indirect lymphography for canine mammary tumors. Animals.

